# Supplementary material for: Fermented Maillard Reaction Products by Lactobacillus gasseri 4M13 Alters the Intestinal Microbiota and Improves Dysfunction in Type 2 Diabetic Mice with Colitis
Source: Pharmaceuticals (Basel). 2021 Mar 28;14(4):299. doi: 10.3390/ph14040299 (PMC8066505; doi:10.3390/ph14040299)
Supplement: Supplementary file 1 [file pharmaceuticals-14-00299-s001.pdf]

Figure S1.

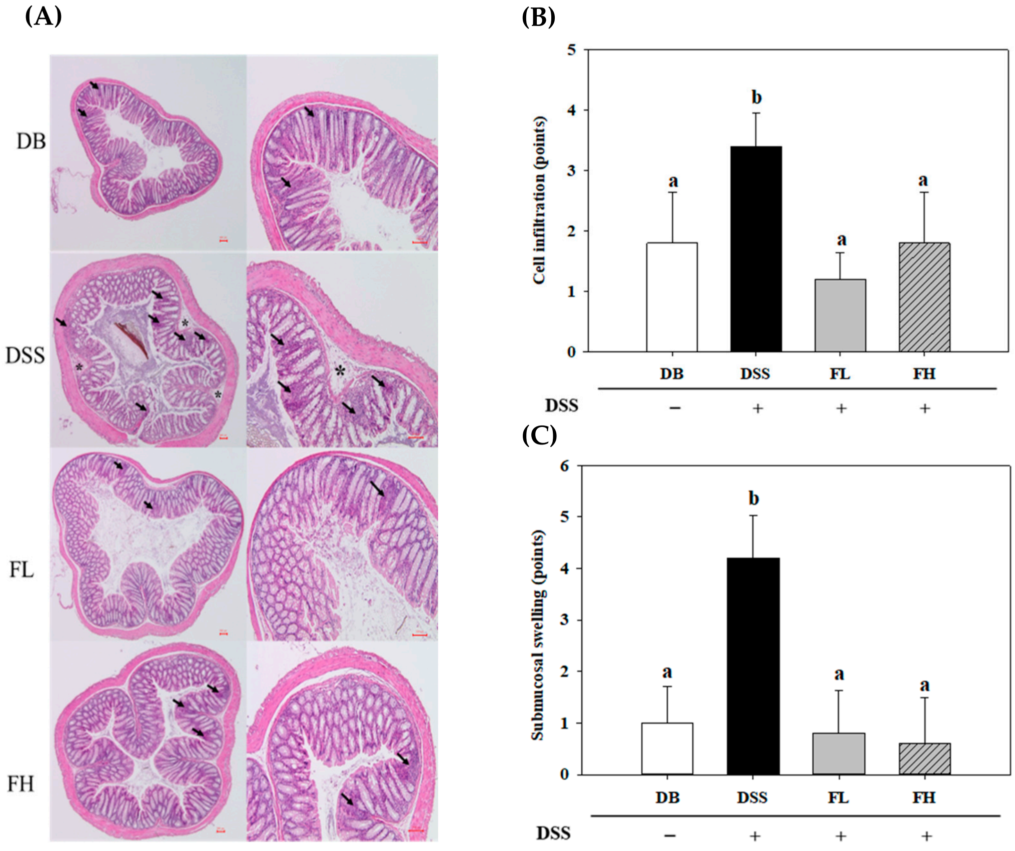

Figure S1. Histological changes in the colon following FMRP treatment. Hematoxylin and eosin (H&E)-stained photomicrographs showing the colon (×200 and ×400 Bar = 100 μm). Morphological changes were visualized using a light microscope after H&E staining (→: cell infiltration, *: submucosal swelling). Different letters (a–b) denote a significant difference at $p < 0.05$ calculated using one-way ANOVA with Tukey's multiple comparison test. Data are expressed as means ± SD. Five mice were included per group.

Figure S2.

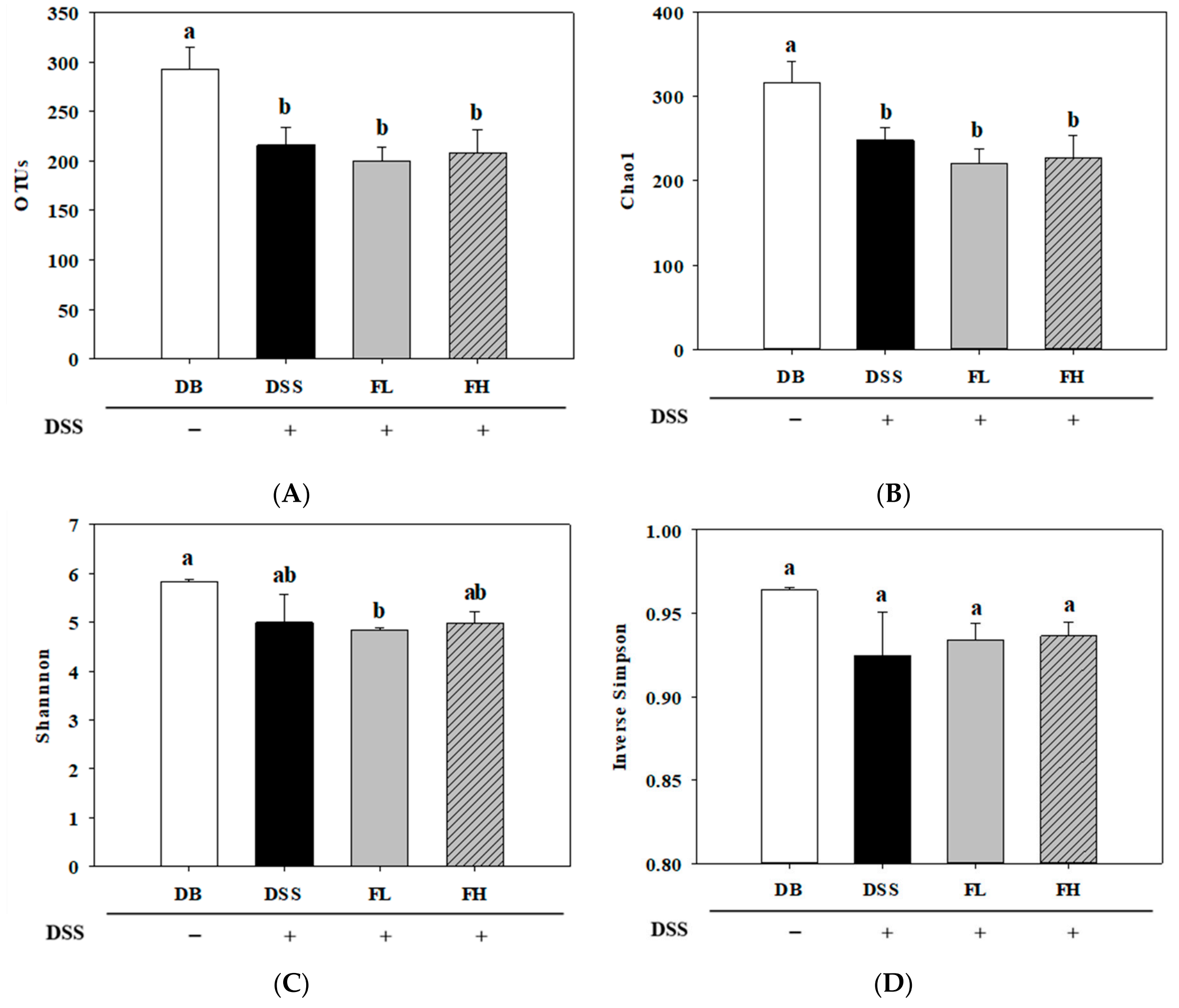

Figure S2. Microbial community analysis of cecal samples in colitis induced DB/DB mice. Alpha diversity indexes are composite indexes reflecting abundance and consistency. (A) Operational taxonomic unit (OTU) levels, (B) Chao1, (C) Shannon's and (D) Inverse Simpson indices. The different letters (a–b) denote a significant difference at $p < 0.05$ using a one-way ANOVA with Tukey's multiple comparison test. Data are expressed as means ± SD. Three cages (3 mice/cage) were included per group.

Figure S3.

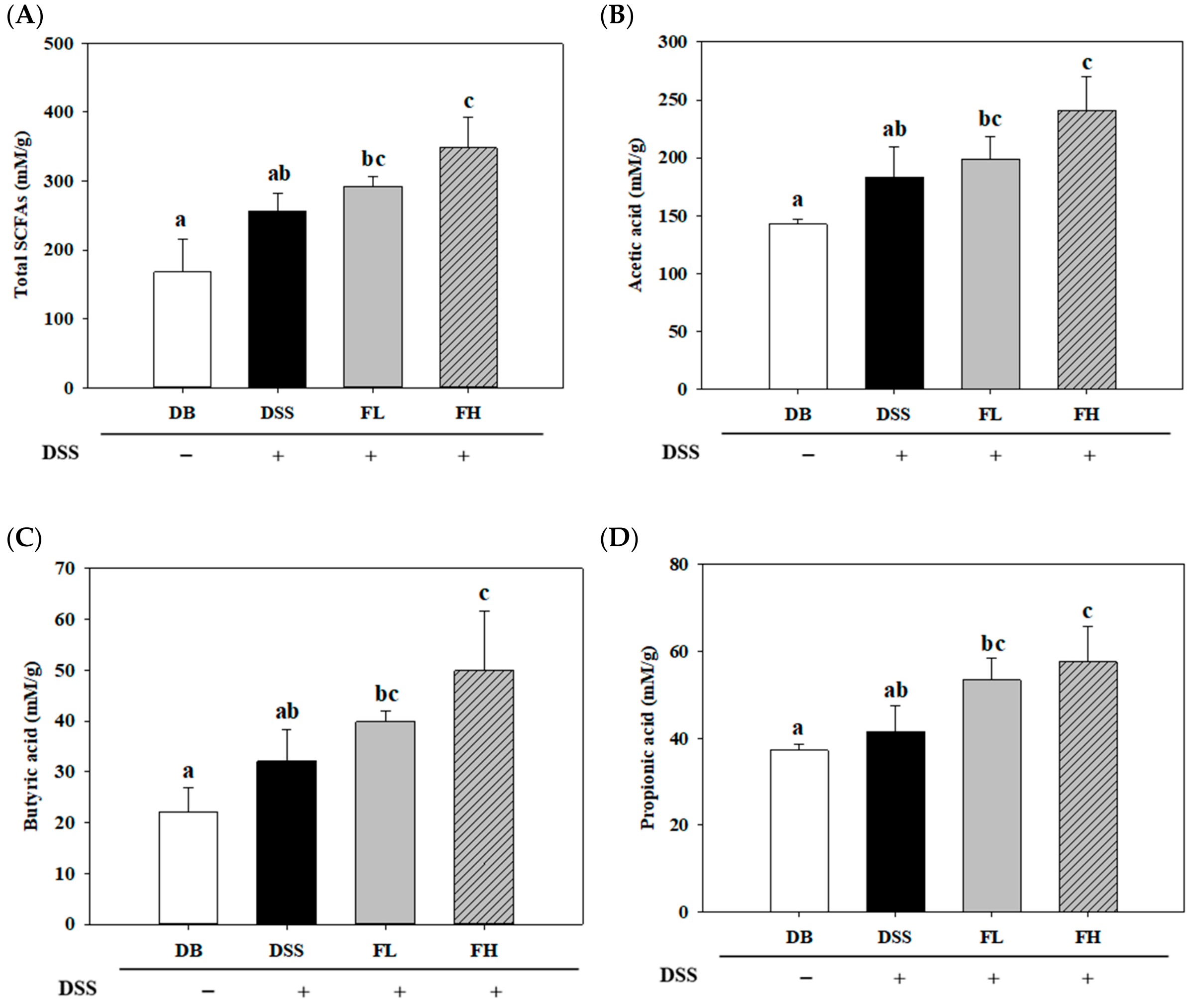

Figure S3. Levels of short-chain fatty acids (SCFAs) in the cecal contents. (A) Total SCFAs, (B) acetic acid, (C) butyric acid, and (D) propionic acid were analyzed using GC-MS. Different letters (a–c) denote a significant difference at $p < 0.05$ calculated using one-way ANOVA with Tukey's multiple comparison test. Data are expressed as means ± SD. Five mice were included per group.
